# Optimization of the Production Factors of Boards Obtained from *Arundo donax* L. Fibers without Added Adhesives

**DOI:** 10.3390/molecules25071660

**Published:** 2020-04-03

**Authors:** Diego Ramos, Francesc Ferrando, Xavier Farriol, Joan Salvadó

**Affiliations:** 1 Mechanical Engineering Department, Universitat Rovira i Virgili, 43003 Tarragona, Spain; diego.ramos@urv.cat (D.R.); f.ferrando@urv.cat (F.F.); 2 Chemical Engineering Department, Universitat Rovira i Virgili, 43003 Tarragona, Spain; xavier.farriol@urv.cat

**Keywords:** *Arundo donax* L., fiberboard, lignin, MOE, MOR, IB, TS, water absorption (WA), steam explosion (STEX)

## Abstract

The main objective of this work was to further analyze the optimization of the production factors of *Arundo donax* L. fiberboards obtained without adhesives. The production of boards derived from *Arundo donax* L. without added adhesives and with high mechanical performance has already been demonstrated. This present study explored a modification in the production process through a final curing thermal treatment (final heat treatment, FHT). Since pressing time is an influential factor in the production cost, it is expected that curing allows a reduction of this time. This study compared the results obtained by three panel-production alternatives: long pressing time (tp) without curing and long and short tp with FHT. Of the two factors analyzed, pressing pressure (Pp) was the most important production factor in both the modulus of elasticity (MOE) and modulus of rupture (MOR), while curing was the most important factor for the internal bond (IB). The study shows that a FHT facilitates the distribution of lignin and a possible improvement in the quantity and quality of bonds between lignin and cellulosic fibers. As a consequence, it improves the IB, produces boards with more homogeneous physical and mechanical properties and thereby makes them more hydrophobic. The curing thermal treatment allows high performance panels to be obtained in a manner which is more ecological, quicker, and cheaper.

## 1. Introduction

*Arundo donax* L. (hereafter: AD) is a material that is widely available in warm areas throughout the world. According to recent studies, the characteristics of this material make it a good choice as feedstock and for obtaining products of relatively high added value [1]. As for the environmental benefits of AD, its carbon storage capacity has been assessed under different fertilization ratios [2].

Not all is positive, however, because of the damaging effects of the invasion of AD on the biodiversity of the riparian areas of several Spanish rivers. This has been especially detrimental to the native flora and fauna of arthropods in the soils of AD [3]. Its distribution and threat as an invasive plant in European continental and Mediterranean waters have also been studied [4], as too have been the agronomic and environmental impacts of this cane in mountainous areas subject to soil erosion [5].

The possibilities of producing biomass from AD in marginal lands of the Mediterranean have also been studied and evaluated [6,7]. To this effect, the impact of salinity and water stress on the production of biomass from AD [8] and also how manure and nitrogen contribute towards its production have been important issues [9].

Further studies on AD have assessed how optimal and justifiable its production in southern Europe would be, in economic terms [10]. In Costa Rica, studies have followed the behavior of AD pellets, which have been produced in different agroclimatic conditions [11]. In this respect, the capture of heavy metals in agricultural soils and wastewater [12] and the elimination of copper in different ecotypes is extremely important [13]. The yield of dry biomass and the composition of carbohydrates and lignin have been quantified in order to determine its bioenergetic potential. [14].

The physical and mechanical characteristics of the AD particle board based on urea-formaldehyde resin have been assessed [15,16,17], whereas the results of other boards without the contribution of any adhesive have been obtained [1,18,19].

The hydrolysis of *Arundo donax L.* L. has also been considered for the synthesis of flexible polyurethane foams [20]. Hydrolyzed A. donax has been used for the production of organic acids of a biological basis as an ecological alternative to petroleum products [21]. The production of cellulosic ethanol from pretreated Aruno donax has also been considered [22].

The acceleration of climate change implies an ever-increasing concern about the consumption of fossil fuels as well as the use of petroleum-based materials. This has become an important incentive for the investigation of materials derived from renewable sources, such as boards obtained from lignocellulosic materials, or even more interesting, the production of boards without any kind of adhesive at all. Such fiberboards have already been demonstrated as being a valid option [1].

The production of boards with these materials involves the fixation of atmospheric CO_2_ for a period of time, which may be important for the environmental advantages that could imply. The construction of furniture and buildings with lignocellulosic materials is socially more beneficial as regards this important fixation of CO_2_, when compared to constructions with other materials such as concrete or ceramics, with significant carbon footprints.

Another fundamental aspect is the limitation of formaldehyde emissions that commercial panels must comply with, especially because restrictions are increasing. It is therefore extremely beneficial to achieve panels without the risk of formaldehyde emissions, an essential component of most of these synthetic adhesives.

From a technical point of view, a major problem with fiberboards is achieving high internal bond (IB) values. This is the most limiting mechanical parameter of fiberboards. The IB is highly conditioned by the uniformity of the lignin redistribution between the fibers and, in turn, the distribution of lignin is believed to depend on the temperature reached inside the boards, as well as the time spent in the application of heat. The outer faces of the boards are always more “toasted” and there is a better distribution of lignin in those external layers. In the inner layers of the board, the lignin remains less “cooked” and its adhesive function is less effective.

There are studies on the influence of temperature on the formation of furfural and 5-hydroxymethyl furfural when hot-pressing lignocellulosic materials [23]. These compounds can increase crosslinking bonds between fibers and improve the internal bond [24].

The above studies invited us to explore the effects of final heat treatment (FHT), which requires keeping the formed boards for a period of time at a temperature higher than the lignin melting temperature. Thus, the boards are expected to establish more and better links with the cellulose fibers, to have better IB, to be more hydrophobic, and to have more homogeneous properties. This will also allow the pressing time to be reduced, which could result in an improvement in industrial productivity and also a lower cost of production.

## 2. Results and Discussion

### 2.1. Series 11 and 21: Comparison of Production Process with and without Final Heat Treatment (FHT)

With this series of experiments our aim was to determine the effect of the application of a final heat treatment (FHT) on the physical and mechanical characteristics of the boards to which it was applied.

Both series 11 and 21 consisted of eight repetitions of the same board. Both series were carried out with the same material and the same process and conditions of elaboration and pressing. The difference is that the 21 series was subjected to a final heat treatment of 5 h in a stove at 165 °C (see Table 1).

The results and the parameters that allowed for statistical analysis are presented in Table 2.

All of the studied characteristics presented statistically significant differences.

**Figure 1:**
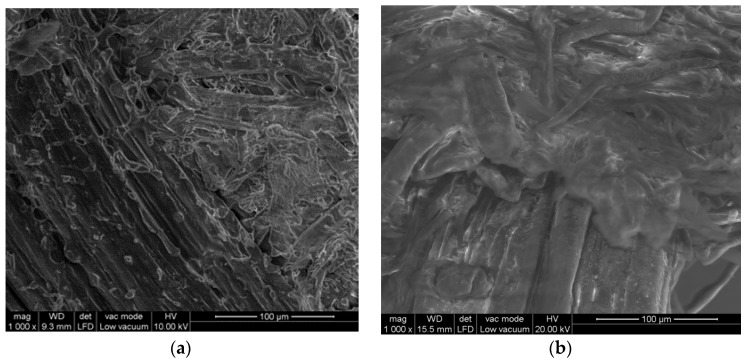
Microscopic images. Material with (**a**) and without (**b**) final heat treatment.

Observing the result of the separation of averages, we found that the density of the 21 series panels was statistically higher than that of the 11 series, although we were at the limit of this difference being significant. The modulus of elasticity (MOE) of series 21 had lower mean values, although we were well above what is required by the EN 622-5:2006 (2007) [26] for structural use boards. There was an important decrease of the standard deviation in the series 21 (S21). The modulus of rupture (MOR) responded in a similar way to the MOE with lower mean values (S21) but with less deviation between them, which indicates an improvement in the uniformity of these boards. The fact that the MOE and MOR were lower in Series 21 can be justified by a possible degradation of the fiber by the continuous and prolonged effect of high temperatures in the FHT, as was previously suggested [27]. We observed that the FHT produced an improvement in the IB of the boards. This may be due to a better redistribution of the lignin between the fibers (see Figure 1) and to an increase of the lignin–fiber–lignin bonds possibly due to a furfural production [23], which generates improvements in the IB [24]. The swelling of the boards decreased with the final heat treatment, which improved their stability.

Water absorption (WA) met the same criteria as thickness swelling (TS), with a significant drop in its average values. This lower water absorption is explained by the higher and better coating of the fibers by the lignin, as can be seen in the microscope photographs (Figure 1). This may be due to the redistribution of lignin during the FHT, which causes a waterproofing effect of the fibers.

The variances are smaller in the 21 series for MOE, MOR, TS, and WA. These results indicate an improvement in the uniformity of the properties of the boards.

Bearing in mind that the FHT represents an important improvement in the most critical parameter, IB, the application of the final heat treatment is very convenient. The obtained boards exceed the normative quality parameters. This makes it possible to reduce the pressure and pressing time and therefore lower the industrial production costs.

### 2.2. Press Time Reduction (Series 22).

Without applying FHT, in order to obtain the necessary IB values according to the standard, high pressures and high pressing times are required. The hot pressing time, being a discontinuous phase within the industrial process, limits the productive capacity of the industry. Once the improvement of the IB with the FHT was observed, the aim was to optimize the hot pressing process. The pressing time can be reduced in order to make this phase faster, thus attaining a greater productive capacity for the production plant. At the same time, a lower energy consumption and a higher sustainability in the process can be attained. This will lead to reducing production costs.

Given that the effect of pressing pressure (Pp) is more important at low pressing pressure, we considered the possibility of reducing Pp and then applying the final heat treatment to homogenize the distribution of lignin between the fibrous material.

The hot pressing process in this treatment (series 22), instead of applying two pressing cycles equal in both time and pressure, was as follows: A first pressing of 2 min at 1 MPa (at 200 °C) followed by one minute of decompression and a second pressing action of 30 s at variable pressure to assess its effect on the characteristics of the panels. Further details of the experimental design can be found in Table 3. In Table 4 the values of the different parameters measured for series 22 are presented. Each parameter was analyzed from a statistical point of view.

#### 2.2.1. Density

**Figure 2:**
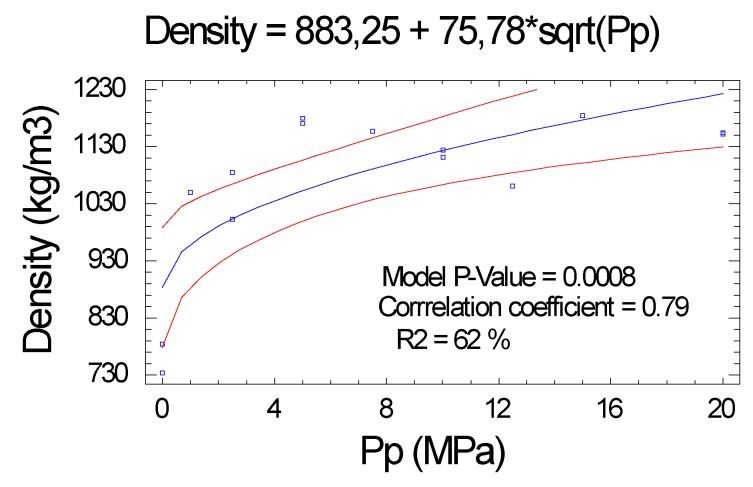
Series 22: Influence of pressing pressure (Pp) on density.

The analysis of regression and variance shows us the relationship between Pp and density, with a correlation coefficient of 0.79, see Figure 2. The proportion of variability of the density values explained by the exposed function was comparatively low (R^2^ = 62%), compared to the explained variability of other characteristics, which is much higher.

#### 2.2.2. Modulus of Elasticity (MOE)

**Figure 3:**
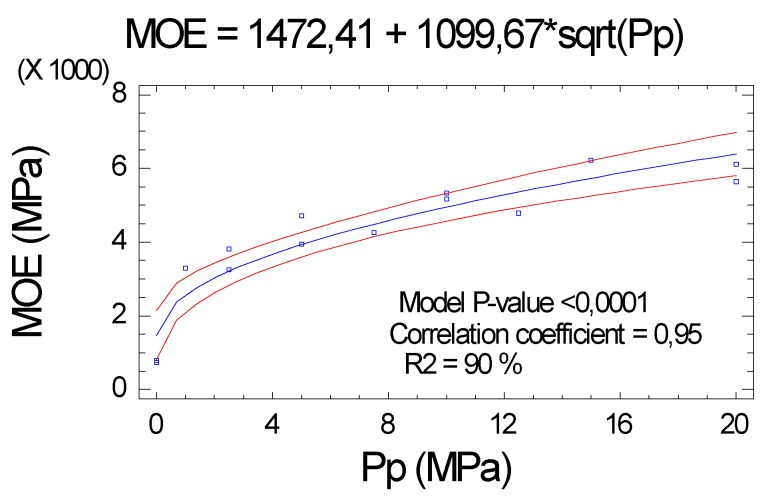
Influence of Pp on the modulus of elasticity (MOE).

The MOE had a very high correlation with the Pp (correlation coefficient of 0.95), with very high confidence levels (*p* value < 0.001) for all parameters of the function, see Figure 3.

#### 2.2.3. Modulus of Rupture (MOR)

**Figure 4:**
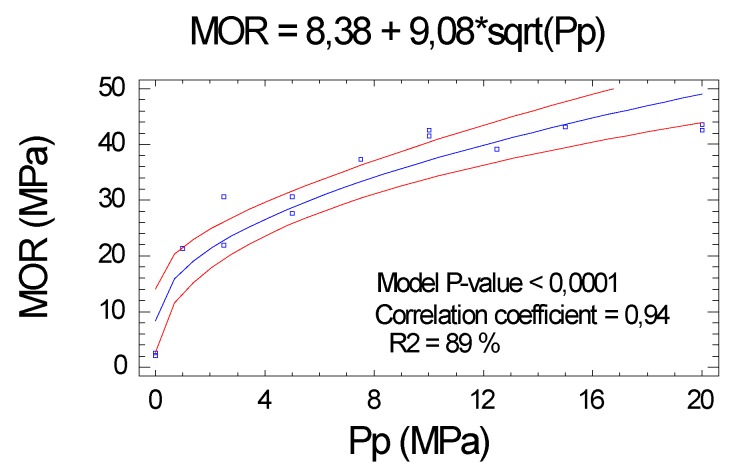
Influence of Pp on the modulus of rupture (MOR).

As in the case of the MOE, the MOR had a very high correlation with the Pp applied to each board and explains the variability of the MOR of the boards (correlation coefficient of 0.94 and R^2^ = 88.7%), including a high level of significance (*p* value < 0.01), both for the ordinate, slope, and model as a whole, see Figure 4. 

#### 2.2.4. Internal Bond (IB)

**Figure 5:**
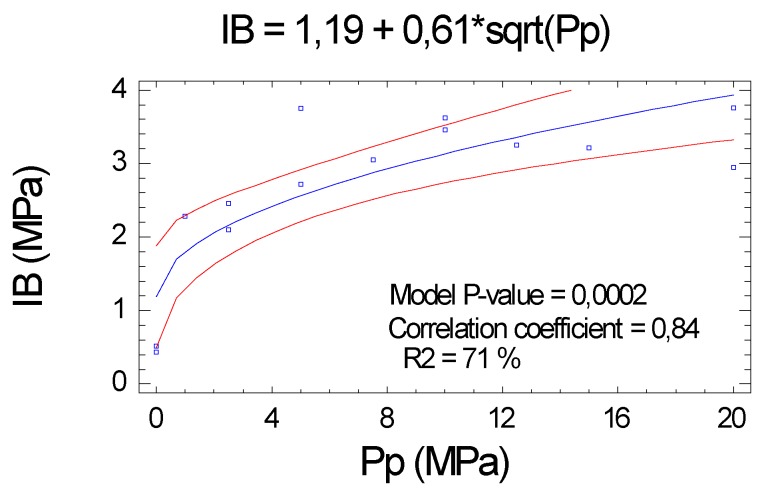
Influence of Pp on the internal bond (IB).

IB also had a high correlation with Pp, and its variability is mostly explained by the proposed function (correlation coefficient of 0.84 and R^2^ = 71.1 %). The level of significance was slightly lower than those obtained for the MOE and MOR, but still remained very high (*p* value <0.01), see Figure 5.

#### 2.2.5. Thickness Swelling (TS)

**Figure 6:**
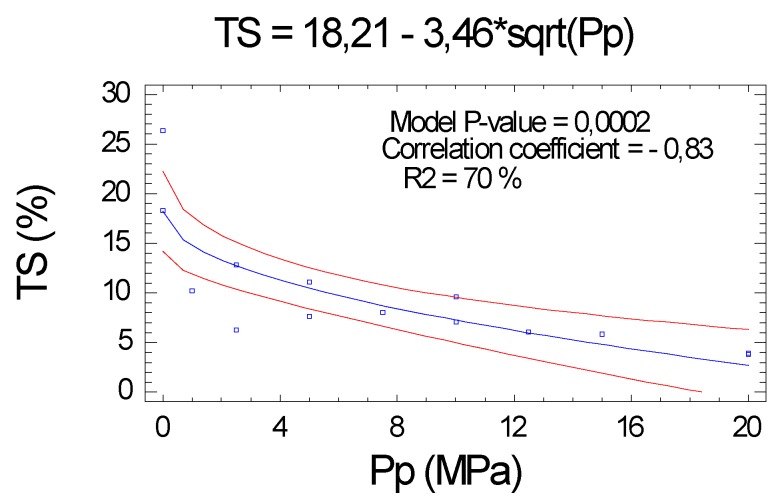
Influence of Pp on the thickness swelling (TS).

We conclude that the swelling is clearly related to the Pp (correlation coefficient of −0.83). The variability explained by the proposed function was around 70%. The level of significance did not decrease with respect to the previous characteristics. As we will see in the different series, as the Pp increased, the swelling of the boards decreased, see Figure 6.

#### 2.2.6. Water Absorption (WA)

**Figure 7:**
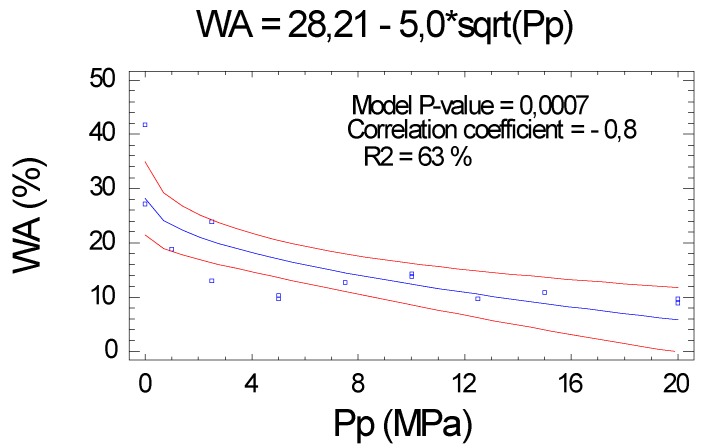
Influence of Pp the water absorption (WA) in function of Pp.

As with the TS, the WA confirmed the negative relationship with the Pp. The correlation obtained between Pp and WA was important (correlation coefficient of −0.8), and the R^2^ was average (63.4%), all with a high level of significance (*p* value < 0.0001), see Figure 7.

The entire series 22, in which the procedure was modified by shortening the pressing time and applying the final heat treatment, had the same type of tendency in its curves: when pressure increased, there was very rapid growth (density, MOE, MOR, IB) or decrement (TS and WA), with much less growth once this faster initial phase had passed. The effect of the pressing pressure was also modelled in this series for all physical and mechanical characteristics. These models can help to optimize the production costs of the panels

The above shows that high mechanical performance panels can be obtained by shortening the hot pressing time, provided that the FHT described in this work is applied.

All the physical and mechanical characteristics of the boards (except for very low Pp) accomplish the specifications required by EN 622-5: 2006 [26] standards for structural use boards.

### 2.3. Complementary Analyses: Comparison of Series 11 and 22 Boards and Series 21 and 22 Boards

In Table 5, the mean values of the physical and mechanical parameters of the boards obtained in series 11, 21, and 22 are shown to allow for a comparison of the effects of pressing time and final heat treatment.

#### 2.3.1. Comparison between Series 11 and 22

Series 22 and 11 panels have the same base materials and pretreatment and start with the same production conditions (pressing temperature 205 °C, pressing pressure 5 MPa). The differences in the process are as follows:

*Pressing Time.* Series 11: 3.75 min (pre-press) + 1 min pressure relief + 3.75 min (final press); Series 22: 2 min (pre-press) + 1 min pressure relief + 0.5 min (final press) and a final heat treatment was provided, as explained in the previous sections.

The comparison of the values of the different parameters is presented below.

*Density.* The values of density were similar in both series.

*MOE and MOR.* Both values were lower in the series 22. The lower value of the MOE and MOR can be explained by the degradation of the fibers caused by the final heating treatment, which involves a long period of time (5 h) at a high temperatures (150 °C). Despite this, the 22 series presents values higher than those required by the UNE Standard for structural use.

*IB.* The internal bond was much higher in series 22 due to the possible “curing of the links” and possibly better distribution of the lignin involved in FHT. It can thus be concluded that IB is much more affected by curing than by pressing time.

*TS.* There was not much difference between the two series. The great dispersion of the measures could be one of the causes for not finding a good explanation.

*WA.* A great difference was observed. The series 11 absorbs much more water. The values of the WA were obtained using a precise weighting method, this provides less dispersion in the results than in those of TS. As in IB, the curing and better lignin distribution allows for better “waterproofing”. Again, a greater effect of the FHT than of the pressing time was observed.

#### 2.3.2. Comparison between series 21 and 22.

The comparison of the values of the different parameters in these series allow us to determine the effect of pressing time.

*Density.* The density was higher in series 21. Higher pressing time results in higher material compaction.

*MOE and MOR.* There were lower values of both parameters in series 22. A lower pressing time provides a looser compaction, which means a weakening of those mechanical parameters. In all cases the values in both series were above the requirements of the UNE standard.

*IB.* The internal bond values were higher in series 21. It is suggested that the longer pressing time at pressing temperature (Tp) (205 °C) in series 21 is the determining effect of the lower IB, due to a possible degradation of bonds at high temperatures. Further research to find the temperature threshold that starts degrading the bonds should be performed.

*TS and WA.* Both values were well beyond the required specifications due to the FHT. In this case, the pressing time did not have a major effect on those parameters. This means that the pressing time can be reduced without decreasing the board quality.

#### 2.3.3. Microscopic Study of Boards made with *Arundo donax L.* L.

In the respective images obtained from the IB fractures, presented in Figure 1, clear differences appear between the boards with and without a final heat treatment. If we look at the micrographs of the untreated boards, they show a more fibrous appearance. Lignin droplets are not observed. On the other hand, the boards with heat treatment clearly show the molten lignin. The plane of fracture in measuring the IB was produced fundamentally by the rupture of the lignin, providing a much higher IB value.

We consider that the final heat treatment is what causes the lignin surrounding the fibers to melt gradually during the 5 h of treatment at 165 °C, allowing for better distribution and the formation a more compact whole.

## 3. Materials and Methods

### 3.1. Materials

The starting material was two-year-old wild reeds collected from the banks of a stream in the area of Tarragona (Spain). The reeds were cut with a GA 100 Black & Decker chipper (Towson, MD, USA). The dry chips were kept in jute sacks to facilitate transpiration and avoid possible fermentation. Immediately following chipping, the humidity of the used material was 54.6% w_water_/w_humid material_. The time between collection and steam explosion (STEX) pretreatment was between 3 and 6 months.

### 3.2. Panel Production Process

**Figure 8:**
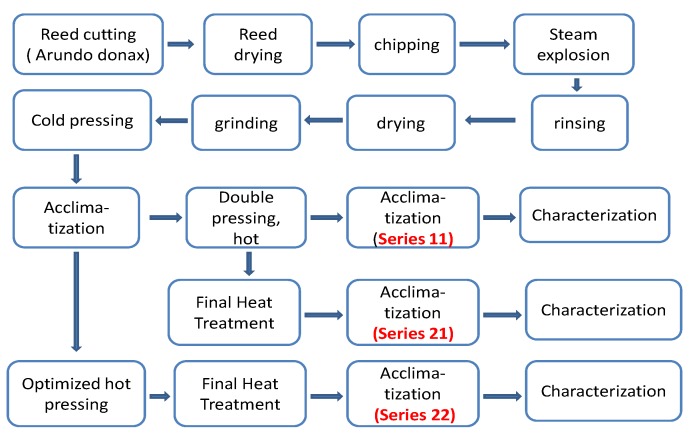
Diagram of production process for boards.

The main production process is shown in Figure 8 and was explained in detail in a previous article [1].

The initial production phase consisted of a hydrolytic steam explosion treatment followed by the sequence: washing, drying, crushing, cold pressing, and conditioning of the moisture of the material in a climatic chamber. From this point onwards, the following series of experiments were designed (see Figure 1).

- Series 11: double hot pressing (see details below) and no final heat treatment.
- Series 21: double hot pressing with final heat treatment.
- Series 22: optimized hot pressing (see details below) with final heat treatment.

#### 3.2.1. Steam Explosion (STEX)

About 800 g of chopped reeds were introduced into a batch reactor (V_reactor_ = 16 L). Saturated steam from a Borealis boiler (Vienna, Austria) of 380 V/82 KW at 40 bar was introduced into the reactor until the required temperature (200 °C) was reached. After the stipulated reaction time (9.5 min), a valve was suddenly opened to connect the reactor with an expansion chamber (V = 100 L), which caused a sudden decompression and the “explosion” of the material.

To define the intensity of this treatment we used a severity factor (Severity) that takes into account the temperature and treatment time [28].

The equation used is as follows:

(1) $$\mathrm{Severity}=\int_{0}^{t\left(min\right)}e^{\frac{T\left(℃\right)-100}{14.75}}dt$$

where T is the steam explosion temperature (°C) and t is the steam explosion time (min).

#### 3.2.2. Washing, Drying, and Grinding of Pretreated Material

The exploded material was rinsed in a washing trolley with a 170 Mesh stainless steel sieve to remove some of the water-soluble hemicelluloses. The washed material was dried to ambient conditions. The dry material was bagged until the next phase (grinding). The pretreated, washed, and dried material was ground in a Retsch SM 100 mill (Düsseldorf, Germany) with a 4 mm sieve.

#### 3.2.3. Cold Pressing and Stabilization at Constant Temperature (T) and Relative Humidity (RH)

For each board, 28.5 g of ground material were taken, in order to obtain a board with dimensions of 150 × 50 mm and approximately 3 mm thickness.

The material, once weighed, was introduced and distributed uniformly in the mold. It was then cold pressed at 16 N/mm^2^ in a conventional press (MEGA-30 AN, Berriz, Spain), leaving a preformed board. These panels were conditioned in the climatic chamber at 20 °C and 65% RH. The panels were kept in these conditions until the next phase, which did not start until the panels maintained a constant weight.

#### 3.2.4. Hot Pressing

The hot pressing was carried out in a heated press (Servitec Poystat 300 S, Wustermark, Germany).

The temperature, pressure, and hot pressing times were regulated in the press. In all the series presented, the pressing temperature was 205 °C. The pressing pressure (Pp) was 5 MPa except for the 22 series, where a pressure range between 0 and 20 MPa was applied. The pressing time in series 11 and 21 was 7.5 min, applied in two stages of 3.75 min, with one minute of decompression between them to facilitate the evacuation of steam. In series 22, a first stage of 2 min was applied at 1 Mpa followed by one minute of decompression and then 30 s at the set pressure.

#### 3.2.5. Final Heat Treatment (FHT)

The boards of the series 21 and 22, once pressed, were introduced into a SELECTA aerated stove of 1600 W, JP Selecta S.A (Abrera, Barcelona), where they were maintained for 5 h at 165 °C.

After the FHT, the boards were kept in the climatic chamber at 20 °C and 65% RH until they were used in the characterization tests.

### 3.3. Experimental Design

An experimental design of one factor (Pp) with different levels was used. In series 22, different levels of hot-pressing pressure were applied to determine the effect of Pp on the diverse characteristics of the panels and to seek optimal results. The statistical treatment was carried out with the support of the Statgraphics Plus 5.1 software, Statpoint Technologies, Inc. (Warrenton, VA, USA).

### 3.4. Characterization of the Boards

The selection of the mechanical characteristics tested was carried out in accordance with the European Normative EN 316: 1999 [29]. The standard EN 622-5: 2006 [26] establishes the requirements for fiberboards made by dry process (MDF) for general and structural use in dry environments. The modulus of elasticity (MOE) and the modulus of rupture (MOR) were tested in accordance with EN 310 [30], internal bond (IB) in accordance with EN 319 [31], and TS in accordance with EN 317 [32]. For the definition of the dimensions of the specimens (150 mm long × 50 mm wide), the criteria established by EN 325: 1993 [33] was followed. For the characterization of the panels, the specimens conditioned in the climatic chamber at 20 °C and 65% RH up to constant mass were used. Constant mass was considered as when the results of two consecutive weighings, carried out in an interval of 24 h, differed by less than 0.1%. The calculation of MOE, MOR, and IB was carried out on a universal testing machine HOUNSFIELD H10KS. For the calculation of TS and WA, specimens were immersed in a water bath with a pH of 7± 1 and a temperature of 20 ± 1 °C for 24 h. After this time they were removed from the water, the excess water was removed, and the boards were weighed and measured again. The scales had an accuracy of at least 0.01 g. In this case, an HM-120 balance was used. The WA is defined as the water absorbed under these conditions as a percentage of the weight of the original dry specimen. The thickness was measured with a digital micrometer (Mitutoyo 547-400S, Kawasaki, Japan).

The SEM pictures were obtained using a JSM 6400. The samples were gold coated prior to the scanning electron microscopy

## 4. Conclusions

The FHT slightly decreased the MOE and MOR of the boards, possibly due to the degradation of the fibers when overexposed to high temperatures for a long time.

The FHT especially improved the IB, TS, and WA thanks to the redistribution of the lignin and the increase in quantity and quality of the lignin–fiber bonds generated by the heat treatment.

It was shown that by applying FHT, it is possible to reduce hot pressing time and obtain boards with high physical and mechanical performance, which allows an increase in industrial productivity and thus reduces costs. At this low pressing time, increasing Pp improves the properties of the panels.

Microscopic images confirm the redistribution of lignin produced during FHT.

The FHT allows us to obtain high performance boards in a more ecological, quicker, and cheaper manner. This kind of material can be used as building material or in the furniture industry.

## Figures and Tables

**Table 1:** Parameters for production of series 11 and 21 fiberboards.

Series	11 and 21	Hot Pressing	2 × tp
Tr (°C)	200	tp (min)	3.75
t_r (min)	9.5	Tp (°C)	205
Severity	8358	Pp (MPa)	5
Washing	Yes	FHT Series 11	No treatment
Cold pressing	16 MPa	FHT Series 21	5 h/165 °C

**Table 2:** Difference of the characteristics of the boards between the series 11 and 21.

Factor	Series	Mean	Standard Deviation	Difference (11–21)	Limits (LSD) +/−
Density * (kg/m^3^)	11	1180	28	−33.2 **	32.21
	21	1213	32		
MOE (MPa)	11	6249	505	1014 *	481.52
	21	5234	385		
MOR (MPa)	11	40.38	3.87	9.21 *	4.08
	21	31.17	3.73		
IB (MPa)	11	1.46	0.51	−1.03 *	0.64
	21	2.50	0.67		
TS (%)	11	11.11	2.53	5.61 *	3.07
	21	5.50	3.15		
WA (%)	11	21.2	2.82	12.05 *	2.54
	21	9.15	1.80		

* Following EN 323-1993 [25]; ** Significantly different following least square difference (LSD) (95%) methodology. Row data found in [18].

**Table 3:** Conditions of series 22.

Series	22	Hot Pressing	2′ at 1 MPa + tp at Pp
Tr (°C)	200	tp (s)	30 except blank (0)
t_r (min)	9.5	Tp (°C)	205
Severity	8358	Pp (MPa)	variable
Washing	Yes	FHT	5 h/165 °C
Cold pressing	dry		

**Table 4:** Results for series 22.

Board	Pp	MOE	MOR	IB	Density	TS	WA
	MPa	>3000 MPa	>29 MPa	>0.7 MPa	>800 kg/m^3^	<35%	<30%
1 (blank)	0	740	2.072	0.51	734	26.34	41.77
2	1	3296	21.36	2.28	1050	10.22	18.82
3	2.5	3817	30.63	2.10	1084	6.25	13.04
4	5	4711	30.63	2.72	1179	7.64	9.68
5	7.5	4251	37.27	3.05	1157	8.03	12.64
6	10	5323	42.59	3.46	1124	7.07	14.29
7	12.5	4792	39.13	3.25	1061	6.02	9.68
8	15	6222	43.09	3.21	1184	5.84	10.87
9	20	6106	43.61	2.95	1152	3.96	8.99
10	0	795	2.55	0.43	784	18.30	27.17
11	25	3254	21.94	2.46	1003	12.80	23.81
12	5	3950	27.64	3.75	1086	11.13	10.34
13	10	5167	41.57	3.62	1111	9.62	13.79
14	20	5634	42.6	3.76	1154	3.81	9.78

**Table 5:** Results that allow for a comparison between series 11, 21, and 22.

Series	Density *	MOE *	MOR *	IB *	TS *	WA *
Specifications UNE EN 622-5: 2006	>800 kg/m^3^	>3000 MPa	>29 MPa	>0.7 MPa	<35%	<30%
11	1180	6249	40.4	1.5	11.1	21.2
21	1213	5234	31.2	2.5	5.5	9.1
22	1133	4331	29.1	3.2	9.4	10.0

* Mean value.

## Abbreviations

FHT	Final Heat Treatment
IB	Internal Bond, MPa
MOE	Modulus of Elasticity, MPa
MOR	Modulus of Rupture, MPa
Pp	Pressing Pressure, MPa
STEX	Steam Explosion
tp	Pressing Time, min
Tp	Pressing Temperature, °C
t_r	Pretreatment (Steam Explosion) Time, min
Tr	Pretreatment (Steam Explosion) Temperature, °C
TS	Thickness Swelling, %
WA	Water Absorption, %
